# Role of Endoplasmic Reticulum Stress Sensor IRE1α in Cellular Physiology, Calcium, ROS Signaling, and Metaflammation

**DOI:** 10.3390/cells9051160

**Published:** 2020-05-08

**Authors:** Thoufiqul Alam Riaz, Raghu Patil Junjappa, Mallikarjun Handigund, Jannatul Ferdous, Hyung-Ryong Kim, Han-Jung Chae

**Affiliations:** 1 Department of Pharmacology, School of Medicine, Institute of New Drug Development, Jeonbuk National University, Jeonju 54907, Korea; toufiqul_t@yahoo.com (T.A.R.); raghupatilj@gmail.com (R.P.J.); 2 Department of Laboratory Medicine, Jeonbuk National University, Medical School, Jeonju 54907, Korea; ecoarjun156@gmail.com; 3 Department of Radiology and Research Institute of Clinical Medicine of Jeonbuk National University, Biomedical Research Institute of Jeonbuk National University Hospital, Jeonju 54907, Korea; jannatulferdous2031@gmail.com; 4 College of Dentistry, Dankook University, Cheonan 31116, Korea

**Keywords:** endoplasmic reticulum stress, IRE1α, insulin resistance, calcium, ROS, type 2 diabetes, obesity, metaflammation

## Abstract

Inositol-requiring transmembrane kinase endoribonuclease-1α (IRE1α) is the most prominent and evolutionarily conserved unfolded protein response (UPR) signal transducer during endoplasmic reticulum functional upset (ER stress). A IRE1α signal pathway arbitrates yin and yang of cellular fate in objectionable conditions. It plays several roles in fundamental cellular physiology as well as in several pathological conditions such as diabetes, obesity, inflammation, cancer, neurodegeneration, and in many other diseases. Thus, further understanding of its molecular structure and mechanism of action during different cell insults helps in designing and developing better therapeutic strategies for the above-mentioned chronic diseases. In this review, recent insights into structure and mechanism of activation of IRE1α along with its complex regulating network were discussed in relation to their basic cellular physiological function. Addressing different binding partners that can modulate IRE1α function, UPRosome triggers different downstream pathways depending on the cellular backdrop. Furthermore, IRE1α are in normal cell activities outside the dominion of ER stress and activities under the weather of inflammation, diabetes, and obesity-related metaflammation. Thus, IRE1 as an ER stress sensor needs to be understood from a wider perspective for comprehensive functional meaning, which facilitates us with assembling future needs and therapeutic benefits.

## 1. Introduction

IRE1/ERN1 (Inositol-Requiring Enzyme 1/Endoplasmic Reticulum to Nucleus 1) is the most evolutionarily conserved endoplasmic reticulum membrane resident protein. It is involved in multiple cellular processes and regulates both cell survival and cell death. IRE1, a transmembrane protein kinase gene, was first detected in yeasts while exploring genes involved in the metabolism of inositol phospholipids to complement exogenous inositol for the growth of yeast mutants in which the disruption of the IRE1 locus triggered myo-inositol auxotrophy [1]. Following Peter Walter and Mori K’s benchmark study, IRE1 was identified as a UPR molecule on the screen of yeast genes involved in signal transduction from the endoplasmic reticulum (ER) to nucleus during misfolded protein accumulation/ER stress [2,3]. In yeasts, IRE1 is the sole UPR sensor which governs the response to ER stress [4]. In metazoans, IRE1 is one of the three distinct UPR sensors, and it exists in two isoforms IRE1α/ERN1 and IRE1β/ERN2. IRE1α is ubiquitously present, whereas IRE1β’s presence is restricted to intestinal epithelial cells [5] and airway mucous cells [6]. IRE1α and IRE1β differ in luminal domain amino acid sequences that are not conserved, especially in association with binding immunoglobulin protein (BiP) [7]. Both are functionally different in substrate specificity by their RNase domain [8]. Therefore, this clearly indicates that sensing and activation of IRE1α and IRE1β are different from each other. Moreover, unlike IRE1α, the IRE1β activity is more similar to yeast IRE1 homologue. The amino acid sequence of the human IRE1α and IRE1β sensor, kinase, and RNase domains has 48%, 80%, and 61% identity, respectively [9]. IRE1α activates the X-box binding protein 1 (XBP1) transcription factor through an unconventional splicing event while IRE1β partially reduces the site-specific 28sRNA cleavage translation [9] and also cleaves XBP1 [10].

This difference in the nature of activity would contribute to their different downstream effects. However, the question is how this functional difference is relevant in physiological conditions and why these sensors act differently. The answer could be the tissue environment, intrinsic molecular factors, or the nature of stress. Another point is that, in tissues like the gastrointestinal tract and airway mucous layer, where both isoforms are expressed, the physiological requirement of both the isoforms in these tissues needs to be understood. Both isoforms might function competitively or complimentarily to each other during the UPR induction. It would be interesting to understand the x-factor, which influences the IRE1β expression or repression.

IRE1 functional dimensions are very diverse; however, it has been majorly implicated in ER stress. The tissue, pathological attributes, stress intensity, and the UProsome molecules association/dissociation decide the nature of IRE1 activity. This versatile ER membrane molecule controls various cellular functions, including cell morphogenesis, signal transduction, secretion, and regulation of many chronic diseases. IRE1 expression in cells must be stringently regulated because overexpression and prolonged activation of mammalian IRE1α and IRE1β induce apoptosis [11]. Therefore, during adaptable disturbances, it gets transiently activated and then gets inactivated, whereas in severe stress, its activity is for longer periods, which triggers the apoptosis inducing molecule and results in cell death. The mechanisms of differential regulation of IRE1α in physiological conditions and in different stress levels are still vague. However, this diverse activity is coordinated by a number of molecules from the ER lumen, cytoplasm, and ER membrane, which form the UPRosome. Orchestrating this molecule, cells can be directed towards survival or death. This difference in the nature of activity contributes to their different downstream effects.

ER performs various cellular functions, such as protein folding, post- translational modifications, fatty acid and sterol biosynthesis, xenobiotic detoxification, and intracellular calcium storage [12]. The rough endoplasmic reticulum on its external surface is lined with ribosomes and is involved in processing and sorting of proteins. If the ribosomes translate the mRNA, a synthesized peptide is inserted into the ER according to the signal sequence. Then, the signal sequence is cleaved, and the protein is released into the lumen of the ER. The protein released into the ER may stay in the ER or move through the Golgi to the lysosome or plasma membrane or may be secreted. However, regardless of its final destination, the protein can undergo different processes in the ER lumen. These involve folding, assembling into multisubunit complexes, formation of disulfide bonds, glycosylation, and glycolipid additions. About one-third of total cellular proteins contains secretory proteins, and transmembrane proteins are matured in the ER. Its functions require the environment in the ER to be oxidative and rich in calcium and other protein folding machinery. The protein folding requirement and amount of secretory protein synthesis vary depending on the cell types. Cells which are meant for the secretory functions are rich in ER to meet the demand. Secretory proteins, helped by chaperones and other movements, fold precisely to their native configuration as they pass through the ER. However, cells can encounter conditions in which demand for ER protein folding activities exceeds the efficiency. Subsequently, ER protein folding functions will get a hit by different perturbations like viral infections, cancers, neurodegenerative diseases, diabetes, inflammation, protein-folding diseases, and other aberrations at a cellular level. This results in the accumulation of unfolded proteins in the endoplasmic reticulum, referred to as ER stress. However, the cell has evolved a mechanism to detect these changes and to restore homeostasis by activating signal transducing pathways, known as the UPR, and this process is conserved from yeast to human. Initially, the UPR system attempts to restore homeostasis by inducing transcription of folding enzymes, chaperones, oxidoreductases, and decreasing protein translation, autophagy, lipid biogenesis, vesicular trafficking, and also by degrading ER-associated mRNA, which helps to minimize translation in the initial adaptive phase. However, in the event of failure of this adaptive process due to prolonged stress, UPR triggers cellular apoptotic pathways to remove ER-stressed cells as a physiological process, but unrestricted apoptosis becomes pathological, which in turn leads to loss of cells in essential organs [13]. Thus, the UPR is an essential fundamental process in the quality control of proteins not only during ER stress, but also in normal growth conditions [14].

This review is focused primarily on recent insights/developments in structure, mode of activation, dimer/tetramer/oligomerization, phosphorylation status, partners/regulators, and nuclease activity of human IRE1α. Furthermore, it includes IRE1α involvement in cellular signaling, UPR-dependent, and independent mechanisms as well as its biological meaning in diseases.

## 2. IRE1α: Structure and Mode of Activation

Human IRE1α is a 977 amino acid protein of ~110 kDa. It is located on the ER membrane and consists of an ER luminal domain, a type I transmembrane domain, and a dual enzymatic, hydrophilic, cytosolic C-kinase, and endoribonuclease function domain [4]. The luminal domain comprises 441 amino acids. The important structural and functional necessary amino acids are Cys 109, Cys 148, and Cys 332. Among these, Cys 109 and Cys 148 are conserved, and N-linked glycosylation site exists at Asp-176. The core human IRE1α luminal domain exists between S24-V390 amino acids, where ER chaperone BiP binds [15,16]. However, neither the N-linked glycosylation sites nor the cysteines appear to influence IRE1α activity [17]. The cytoplasmic portion of IRE1α consists of about 512 amino acids, and it has been subdivided into linker, kinase, and ribonuclease based on the function. The amino acid region between 551–832 is further separated into smaller parts containing diverse functional motifs, including AA 551–650 for the adenosine triphosphate (ATP) binding pocket, AA 651–750 for both the catalytic loop and activation loop, and AA 701–750 for the activation loop. The 551–650 part contains a few preserved residues that are specifically included in an IRE1α kinase domain dimer interface interaction basic for the IRE1α autophosphorylation [18] and essential kinase activity residue K547. The IRE1α cytosolic region has six phosphorylation sites; two at linker region (S551, S562), three at kinase activation loop region (S724, S726, S729), and one at RNase domain region (T973). Phosphorylation is a necessary step for the IRE1α to get enzymatic function. Among the six sites, sites at the activation loop play a very important and necessary role. Mutations of S551, S562, and T973 did not affect the splicing activity. This suggests that these sites may not contribute greatly, but phosphorylation of activation loop residues ser724, ser726, and ser729 contribute, with the greatest contribution from ser724 and ser726. Thus, the activation loop mutant reduced XBP1 splicing and regulated IRE1-dependent decay (RIDD) activity [19]. The kinase phosphorylation responds to the activation state of the RNase, proposing that phosphorylation of the activation loop is a vital step in IRE1α -mediated UPR activation, and this indicates that, by regulating, phosphorylation can control the different enzymatic functions, and it may be possible to differentiate RIDD and XBP1 splicing based on the phosphorylation status. The extent of phosphorylation may decide the IRE1α dimer or oligomer formation or vice-versa so that IRE1α can be guided to distinct downstream activity leading to cell death or survival or phosphorylation may trigger the IRE1α to dimerize or oligomerize in the ER membrane plane by binding unfolded proteins to its UPR sensor domain or by discharging oligomerization-repressive chaperones or both, to permit the trans-autophosphorylation of juxtaposed kinase domains [20,21,22].

An RNase domain activation by kinase domain is also influenced by the pre-binding of cofactors. This association governs the subsequent conformational rearrangement of the RNase domain depending on the chemical properties of bound cofactors. Chemical perturbations of cofactors can repress the conformational phase. The oligomerization of the receptor is affected by the cofactor-induced conformational transition. [23,24], and phosphorylation regulates oligomerization [25].

IRE1α can exist in three physiological forms, an inactive monomeric form bound with BiP at the amino-terminal luminal domain (NLD), and an active dimeric or multimeric form. To understand IRE1α comprehensively, many researchers have been trying to elucidate the structure and mode of activation using both yeast and metazoan IRE1 forms. Even though there are distinct opinions and theories, these studies have shed light on this biologically important molecule. The dumpy ER environment during pathological conditions and also at a low level, the regular physiological conditions lead to the activation of IRE1α [20].

The activation models proposed for human and yeast IRE1 are slightly different. It stays as an inactive monomer during unstressed state due to the binding of ER chaperone glucose-regulated protein 78 (GRP78)/BiP. By modulating the sensitivity and dynamics of IRE1α activity, BiP provides a buffer for inactive IRE1α molecules, which ensures sufficient action to maintain homeostasis in protein folding [26]. As soon as misfolded proteins start accumulating in the ER lumen, in the first step, due to its high affinity towards misfolded proteins, BiP dissociates and frees the IRE1α. In the second step, direct interaction of misfolded proteins with core stress-sensing region (CSSR) of IRE1α (which is prevented during normal state) makes, by conformational change, luminal domain homodimerize or oligomerize depending on the stress intensity [27]. In the third step, dimerized protein autophoshorylates at the cytosolic kinase domain, leading to a conformational change in the C-terminal RNase domain and gaining the endoribonuclease function [7,28,29,30]. Four ligands; ADP, quercetin, SR2+, and Mg2+, are involved in stabilizing the active conformation of IRE1α when BiP is dissociated [31,32].

In yeasts, IRE1 activation is regulated through direct interaction with misfolded proteins, but, later, it is complemented by the BiP dissociation [22,27]. This was evidenced by a study where UPR was attenuated in the BiP overexpression system [33,34]. However, the UPR attenuation in the BiP overexpression system could be due to the increased folding activity, which decreased the ER stress rather than directly inhibiting IRE1α activation [35]. Furthermore, Oikawa et al. added that self-association of core luminal region and BiP dissociation are not sufficient for activation of the IRE1α; thus, another unknown change on the luminal side is crucial for IRE1α activation [36]. Membrane lipid aberrancies are also sensed by IRE1α, but maybe in a different manner [37].

An alternative “BiP-independent” model of UPR activation has been suggested in yeast, which points to a direct role for unfolded proteins in UPR activation. At the dimerization interface, the crystal structure of the IRE1 core luminal domain (residues 114449) enters groove, which looks like the peptide-binding domains of major histocompatibility complexes (MHCs) [38]. Interestingly, misfolded proteins can interact directly in MHC as a grove, and it is a critical driving force for the clustering of IRE1 luminal domains, and this will lead to the closure of cytoplasmic domains, resulting in autophosphorylation and conformational change leading to RNase domain activation and further downstream signaling pathways [22,27,39]. Furthermore, IRE1 does not require any specific consensus sequence, but rather binds to peptides containing basic and hydrophobic residues, usually located in the core of folded proteins, but become exposed in misfolded proteins [22].

In contrast, in humans, BiP-dependent activation exists because this groove is too narrow for peptide binding in IRE1α and also peptide binding to this groove is not required for dimerization [40]. However, a recent study from Karagaz et al. delineated the activation mechanism of IRE1 in both yeasts and mammals. They suggested that the IRE1α can also bind to the unfolded proteins similar to yeasts, based on the amino acid in the peptide, then induce allosteric conformation change, which results in the oligomerization at a conserved region [41].

Once IRE1 luminal domains get activated and dimerized, which bring the cytosolic portion closer, trans- autophosphorylation takes place at kinase domains of the two molecules through the binding of nucleotide. Trans-autophosphorylation results in the conformation change in the kinase domain which further allosterically regulate the positioning of the RNase domain [25] for further oligomerization and its activation. IRE1α oligomerization state and RNase domain activity are affected by the conformation of helix-αC in the kinase domain.

The cytosolic domain is important for clustering of both IRE1α and IRE1β, forming foci upon ER stress. The difference between the two molecules is at the signal transition from monomer to oligomer or vice-versa. IRE1α activation seems quick and transient and attenuates soon after adaptation [9,20,42]. However, the activation of IRE1β is slow and continual to elicit apoptotic cell death [9], as observed in the case of sustained repression of microsomal triglyceride transfer protein (MTP) mRNA [43] and chronic change in intestinal lipid absorption [44,45]. These differences in the nature of activity in IRE1α and IRE1β contribute to their different downstream effects.

## 3. Activation Mechanism of IRE1α during Physiological Stress 

Since prolonged activation of IRE1α causes cell death, activation and inactivation of IRE1α must be properly regulated in the cell. Therefore, during adaptable disturbances, it is transiently activated and then gets inactivated, whereas, in severe stress, its activity is endured for a longer period, triggering apoptosis-inducing molecules, resulting in cell death. The mechanism by which IRE1α is differently regulated in physiological and pathological conditions still needs to be understood.

Unlike yeast IRE1, IRE1α luminal domain is sensitive and is easily triggered by minute changes in the ER lumen. Since IRE1α does not have an intrinsically disordered intramolecularly antagonizing subdomain, Subregion I, like in yeast, which tightly represses the yeast IRE1 activity under conditions of no stress or weak stress [46], and mutation at this site results in constant activation and disturbs the yeast growth. Yeast IRE1 has several homomeric interfaces in its lumen and forms polymer oligomers [38]. On the contrary, IRE1α’s luminal domain has a single interface and forms dimers or small oligomers [16]. In metazoans, activation of PRKR-like endoplasmic reticulum kinase (PERK) is tightly controlled because it carries similar subdomains like in yeast IRE1. This could be the reason that, in metazoans, IRE1α is the first UPR sensor to get activated before PERK.

Furthermore, an alternative mechanism of IRE1α exists, where BiP still binds to activated IRE1α, especially in physiological stress, such as inositol depletion for a prolonged time. Under these conditions, IRE1α may be activated as a homodimer. In physiological and in some persistent low-level ER stress conditions, IRE1α is weakly activated, but it is continuous. This low-level activation may not require cluster formation or dissociation of BiP. Like in yeast IRE1, mutant W426A aborted cluster formation, but formed dimer and it still showed considerable activity, and even some chemical ER stress inducers like dithiothreitol (DTT) showed similar activity [47]. This indicates that, upon physiological stress or in some persistent diseases, IRE1α activity may be controlled in dimer state by its associated molecules, which would disrupt the cluster formation to strive for the cell adaptation rather than apoptosis.

However, this diverse activity is coordinated by the number of molecules from ER lumen, cytoplasm, and ER membrane, which forms the UPRosome. The tissue, pathological attributes, stress intensity, and the UPRosome molecules association or dissociation decide the nature of the IRE1 activity.

## 4. IRE1α in ER Stress and Its Crosstalk with Other UPR Signal Transducers

UPR is mediated by three ER membrane localized sensors IRE1α, PERK, and activating transcription factor 6 (ATF6), which induce different interconnected downstream signaling cascades to influence the life–death decision. However, these transducers are negatively regulated in normal conditions by the ER chaperone BiP/GRP78, but, during ER stress, BiP dissociates and binds to the misfolded proteins. UPR transducers that are free of BiP get activated and trigger downstream signaling pathways that try to reestablish the normal ER function.

The PERK/EIF2AK3 pathway restores the homeostatic condition by reducing the new protein load by attenuating the protein translation. Activated PERK dimerizes and is autophosphorylated and then forms large clusters [28] which phosphorylate eIf2α (eukaryotic translation initiation factor 2 alpha) [48] on Ser51 and inactivate its activity, which results in attenuation of protein synthesis. However, phosphorylated eIf2α can selectively allow the mRNAs with internal entry sites/mRNAs containing short open reading frame (ORF) in their 5’ UTR (µORF) like Activated transcription factor 4(ATF4) [49]. This transcription factor activates both prosurvival genes involved in protein folding, redox metabolism, autophagy along with endoplasmic-reticulum-associated protein degradation (ERAD), and also initiates the expression of apoptotic gene C/EBP homologous protein (CHOP)/GADD153. Furthermore, CHOP induces GADD34 which restores the protein synthesis by dephosphorylating eif2α through interacting with protein phosphatase 1C(PP1C) [50]. A short-time halt in protein translation is advantageous for cell survival, but chronic ER stress PERK signaling upregulates transcription factor ATF4 and CHOP, which enhances protein synthesis and contributes to cell death due to ROS production through ERO1 and ATP depletion [51]. PERK also induces cell death by triggering caspase 8 through death receptor 5 (DR5) [52].

However, the interlink activity with other ER stress transducers is not well established. PERK-mediated phosphorylation of eif2α increased the stability of XBP1s mRNA through translation inhibition [53]. This results in increased XBP1 protein levels and its target genes during the UPR. The hepatocyte-specific deletion of IRE1α in mice resulted in the activation of the UPR–PERK pathway [54,55]. Deprivation of IRE1-XBP1s in acinar cells leads to a sustained activation of PERK/EIF2α/ATF4/CHOP pathway and development of pancreatic pathology [56]. The PERK and IRE1α pathway have the control on DR5 expression but exert opposing effects depending on the stress intensity. IRE1α plays an antiapoptotic role by degrading DR5 mRNA during the initial adaptive process, whereas PERK-mediated CHOP increases the DR5 expression in unmitigated stress [52]. Recently, it was reported that PERK regulates the miRNA cluster formation, which in turn regulates the ATF6 activity and also influences the RIDD activity of IRE1α [57]. IRE1α expression is regulated by the PERK/ATF4 pathway during ER stress [58]. Additionally, IRE1α also suppresses protein synthesis by enhancing the phosphorylation of eif2α through its RIDD activity on CReP/Ppp1r15b mRNA, an eif2α phosphatase, and decreases the stress level in the cell [59]. This interconnection between the UPR molecules show their dependency and complementation in maintaining homeostasis in various diseased conditions and also may contribute in decision-making towards survival or death.

ATF6 is a type II transmembrane protein with two subtypes ATF6α and ATF6β, which upon activation by BiP dissociation translocate to the Golgi compartment where it gets cleaved into N-terminal cytosolic domain P50 (50kDa) by two proteases: serine protease site 1(S1P) and metalloprotease site-2 protease (S2P). The cleaved P50 translocates to the nucleus and binds at CRE and ERSE-1 elements and induces the prosurvival genes BiP, GRP94, XBP1, and also prodeath transcription factor CHOP. However, the contribution of ATF6 in ER homeostasis maintenance is relatively minor as it was demonstrated in ATF6 KO mice, which showed no apparent defects, and its function might be compensated by XBP1[31].

IRE1-mediated splicing can activate the translation of a protease, which subsequently cleaves ATF6 [60]. In support of this hypothesis, Wang et al. demonstrated that the kinase-defective mutant hIRE1α K599A blocks ER-stress-induced activation of ATF6 in mammalian cells, indicating that ATF6 cleavage is downstream of IRE1α signaling [61]. ATF6 and IRE1α synergistically control gene expression of endogenous XBP1s in osteoarthritic cartilage [62]. However, the IRE1α-dependent induction of UPR transcription majorly depends on the ATF6 produced XBP1 [31,63]. This indicates that the interdependency of these molecules is evolutionarily developed to maintain homeostasis.

Upon sensing ER stress, IRE1α, molecules form dimers or oligomers on the ER membrane and subsequently the trans-autophosphorylate [22], which results in the allosteric changes in its conformation and the c-terminal RNase domain, will gain the function [4]. Upon activation, IRE1α cleaves introns from specific mRNA by the unconventional method in the cytoplasm in a spliceosome-independent manner, leading to frameshift and introduction of a new termination codon in coding sequence [64], but it requires the existence of a pair of characteristic stem–loop structures and conserved consensus sequence CNCNNGN (N is any base) sequence in mRNA [10,65]. The specific mRNA targeted in yeasts is the HAC1 and removes a 252-nucleotide intron [66]. In mammalians, XBP1 is targeted and removes a 26-nucleotide intron [67], and in plant (Arabidopsis), bZIP60 mRNA is targeted and removes 23 nucleotides [68]. This cleavage generates a 2′3′-cyclic phosphate at the 3′end of the 5′ exon and a 5′-OH at the 5′ end of the 3′exon. Furthermore, these ends are ligated by tRNA ligases, Rlg1p (cyclic phosphodiesterase, polynucleotide kinase, and RNA ligase) in yeast results in spliced HAC1 (HAC1s) transcript [69]. In metazoans, RNA ligation is mediated by RtcB, generating a stable transcription factor that is spliced XBP1 (XBP1s) [70]. In plants, RLG1 generates spliced bZIP60 [71]. RtcB ligation is cooperated by archease [72,73] in GTP and Mn2+ dependent manner [74,75]. Generated XBP1s induce multiple cell survival factors. Additionally, IRE1 activation causes, other than generating a stable spliced transcription factor like XBP1s, cleavage of other ER-localized mRNAs, leading to their degradation in a process named as Regulated Ire1-Dependent Decay (RIDD) [76]. Virus-induced RIDD activity in neuroblastoma cells (Neuro2a) degraded the host RNA, and helped in viral amplification [77]. In addition, IRE1α-dependent decay of the pro-apoptotic microRNA miR-125a leads to the corresponding increase in the amounts of antiapoptotic Bcl-2 family proteins, inhibiting the cell apoptosis in viral infection [78].

IRE1α induces cell death pathway through various routes by activating different apoptosis-inducing molecules. However, this action of IRE1α is very much controlled or restricted depending on the level of stress or type of stress and also on the type of tissue. IRE1α activity is necessary for the normal life of the cell and also in the stress adaptive process, but when the threshold breakpoint crosses the balance of survival and death signals, IRE1α may start cell downfall signals, and this could be regulated by regulating partner molecules. IRE1α triggers cell death by promoting the intrinsic apoptosis pathway by interacting with a hub of diverse molecules through TNF receptor-associated factor 2 (TRAF2). TRAF2 and apoptosis signaling kinase 1 (Ask1) interact and phosphorylate the c-Jun N-terminal kinase (JNK). Sustained JNK activation by controlling the activity of members of the Bcl-2 family is known to cause apoptosis. Interestingly, IRE1 activation of JNK is also confirmed by receptor-interacting serine/threonine protein kinase 1 (RIPK1) via TNF-independent TNFR1 interaction at the ER membrane [79,80]. RIPK1 and IRE1 association may also promote death receptor-independent caspase-8 activation; consequently, caspase-9 and caspase-3 get activated inducing cell death. Additionally, the IRE1–TRAF2 interaction also promotes NF-κB in TNFR1-dependent manner and is dependent on the autocrine production of TNFα. IRE1 induces apoptosis of hepatocyte in ER stress dependent manner by inhibiting AKT through increasing pleckstrin homology-like domain family A, member 3 (PHLDA3) expression [81]. Phosphorylated JNK stimulates the cytochrome c-mediated apoptotic pathway by phosphorylating different members of Bcl-2 family of proteins [82,83]. IRE1α activates multiple signals via its endonuclease and kinase domains to respond to ER stress. The endonuclease domain of IRE1α promotes splicing of the X-box binding protein 1 (XBP1), encoding mRNA, and regulates the IRE1α-dependent decay of mRNAs, including the DR5 encoding [52]. Mammalian target of rapamycin complex 1 (MTORC1) induces apoptosis under ER stress conditions by suppressing Akt and thereby activating the IRE1-JNK pathway [84]. IRE1α regulates certain cell cycle regulatory gene like cyclin A. It involved proliferation with tight control of a cell cycle in an XBP1 dependent manner [85].

RIDD activity has been also reported as a beneficial process during the initial stage of ER stress. This contributes to the cell adaptive process further by reducing the ER load and helping in recovery. However, under unresolved ER stress, the RIDD process may extend its degradative activity to other essential mRNAs, which creates an imbalance in the anti-apoptotic and pro-apoptotic niche, resulting in cell death. Further degradation of mRNA fragments can induce inflammation [86]. It has also been stated that RIDD contributes to the BID-dependent activation of the mitochondrial apoptotic pathway by degrading miRNA to repress caspase-2 expression and activation [87,88]. 

## 5. IRE1α in Cellular Physiological Function

The diversity among cell types like secretory cells, differentiating cells, metabolizing cells, and their functionality necessitates regular adjustment of their ER capacity. Therefore, UPR signaling is almost certainly used even during normal physiology to adjust the ER function in response to fluctuating demands [89]. During cell differentiation, cells require and produce a large amount of secretory proteins. Thus, cells must therefore increase their secretory machinery to handle the high demand. These physiological processes must be handled optimally to progress in the proper development of tissue. Table 1 describes different functions of IRE1α in cellular physiology.

Being a core molecule in UPR, IRE1α is involved in many basic cellular functions other than its involvement in ER stress signal transduction. Its absence has led to the dysfunction of many cellular signals. It has been majorly implicated in cell differentiation, lipid synthesis, membrane integration, secretion, and metabolic activities. IRE1α knockdown results in the embryonic lethality itself due to a reduction in vascular endothelial growth factor-A, labyrinth dysfunction in the placenta, and fetal liver hypoplasia [90], thus showing it is an essential molecule in the cell. However, conditional knockout or knockdown studies have helped to understand this molecule’s important role in cell physiology.

**Table 1:** Different functions of IRE1α in cellular physiology.

Physiological Role	Mechanism	Model/Tissue Region	References
Tissue growth	Inducing XBP1s dependent function.	Liver	[91]
Lipogenesis	Regulates lipogenic gene expression involved in serum cholesterol triglyceride and free fatty acid synthesis.	Liver	[92]
Secretory function	IRE1 deletion impaired the insulin, saliva, and antibody secretion.	Exocrine glands, plasma cell, pancreatic acinar and β cells, salivary serous tissues	[93,94,95]
Lipid metabolism	IRE1β-mediated RIDD activity on MTP and reduce dyslipidemia.	Mice/Liver	[96,97]
Lipid, glucose, and bile acid metabolism	Deletion of hepatic XBP1 disables the bile acid metabolism in mice.	Liver	[94,98]
Organelle biogenesis and homeostasis	IRE1/XBP1 increases the synthesis of membrane phospholipids, especially in secretory cells and fibroblasts to carry out their huge task to meet the physiological demand.	Endoplasmic reticulum	[99,100,101]
B cell differentiation	XBP1s dependent function, deletion impaired differentiation.	Lymphoid tissue	[102]
Eosinophil differentiation	XBP1s dependent function, deletion impaired differentiation.	myeloid tissue granulocyte	[103]
Embryogenesis	IRE1α, IRE1β function in mesoderm development, XBP1 dependent pathway.	Human/Xenopus laevis. Mesoderm, gut	[104,105,106]
Osteoclastogenesis	IRE1α/XBP1-mediated osteoblast and osteoclast differentiation, induction of bone morphogenetic protein-2 and PTHR.	Osteoblast, Osteoclast	[107,108,109]
Immune cell development	IRE1α/XBP1 functions, deletion impaired antigen presentation to T cells, proliferation, and differentiation. Loss of RIDD and XBP1 causes the cDC1 cell death.	Dendritic cells, Lung and small intestine	[110]
Cell cycle regulation	IRE1α /XBP1 drives cells from G1 to S-phase through regulation of cyclin A1 and D1, promote compensatory proliferation of β-cells.	Pancreatic β cells	[111,112]
Photoreceptor differentiation	IRE1α /RIDD level and increased the delivery of rhodopsin-1 to the rhabdomere. Loss of IRE1α disrupted the rhabdomere morphogenesis and the ER anatomy.	Drosophila compound eye R cells	[113,114]
Chondrocyte differentiation	IRE1α negatively regulates chondrocyte differentiation through inhibition of granulin-epithelin precursor (GEP) and by upregulating parathyroid hormone-related peptide (PTHrP).	Chondrocyte	[109,115]
Dendrite morphogenesis	Perturbation of the IRE1 pathway causes loss of dendritic branches.	Caenorhabditis elegans/neurons	[116,117]
Enterocytes	IRE1β inhibited the differentiation of Caco-2 cells into enterocyte-like cells by suppressing microsomal triglyceride transfer protein (MTP).	Intestine	[43]
Mucous secretion	IRE1β knockout mice are viable, but are more susceptible to colitis. IRE1β is needed to maintain normal transcription rates of mucin genes and genes associated with the development of mucins.	Intestine goblet cells, gut epithelium, airway epithelium	[5,6,118]
Metabolic transformation of cells	IRE1/XBP1 pathway contributes to lipogenic gene expression during locational metabolism and lipid metabolism by controlling liver hormone; fibroblast growth factor 21(FGF21).	Mammary gland, Liver, adipocytes	[119,120,121]
Tissue regeneration	IRE1/XBP1 through direct regulation of transcription factor STAT3.	Mice/hepatocyte	[122]
Hematopoietic cells	IRE1/XBP1 pathway plays a role in cell cycle, differentiation of hematopoietic cell.	Hematopoietic tissue	[123]

## 6. Modulation of IRE1α Downstream Activities toward Divergent Cell Fate

Under physiological and pathological conditions, different magnitudes of IRE1α activity indicate that its selection of downstream substrates, XBP1, other mRNA, miRNA, or JNK. Interestingly, the structure–activity relationship studies demonstrated an allosteric relationship between the kinase and RNase domains of IRE1α, which provided an opportunity to modulate its downstream activities [124,125,126]. Many small chemical molecules have been reported to modulate the RNase activity as kinase inhibitors/ATP-competitive molecules, and type 1 kinase inhibitors like 1NM-PP1, APY29, staurosporine, and sunitinib which inhibit autophosphorylation, but induce an active conformational change in both kinase and RNase activity, and type II kinase inhibitors are Kinase-Inhibiting RNase Attenuators (KIRAs) that allosterically inhibit IRE1α’s RNase activity by breaking oligomers [127]. IRE1α activity can be modulated through inhibition or activation to yield diverse clinical benefits depending on the type and condition of the disease since IRE1α serves both adaptive, pro-survival, and pro-apoptotic activity. Numerous studies have been reported about the application of small chemical modulators in other diseases such as cancer or other diseases [128,129,130]. Under ER stress, optimized application of KIRA, KIRA6 and inhibition of IRE1α promoted cell survival and protected photoreceptor cells while maintaining pancreatic β cells and reducing hyperglycemia in Akita diabetic mice in vivo [25]. Information about different chemical modulators was updated elsewhere [131].

Depending on therapeutic purpose, IRE1α modulators specific to either XBP1 splicing or RIDD behavior may be clinically useful. Autophosphorylation and dimer state for RIDD activity [125], which causes decay of many mRNAs, including those encoding chaperones, result in apoptosis. This is bypassed using chemical modulators to activate the RNase by an alternate mode that enforces XBP1 splicing and averts mRNA decay and apoptosis. Therefore, by controlling kinase domain conformation, IRE1α can be directed towards divergent cell fates during ER stress [125]. Additionally, phosphorylation of the IRE1α proportionately increases the oligomeric state of kinase/RNase subunits, reaching a hyperactive state, and its biological roles switch from adaption to destruction [25]. However, oligomerization can be allosterically forced without phosphorylation [132].

## 7. Intrinsic Modulation of IRE1α by Its Binding Partner and Functional Implication

IRE1α interacts with many other molecules, both in physiological condition or stress condition. Collectively, IREα, with its partner’s complex, is termed as UPRosome. In this complex, some of the partners involved enhanced its functions, and stability and some others reduced them (Table 2). The nature of the interaction between IREα and its partners in the complex is dynamically regulated based on the tissue specificity or on the type of insults [133,134]. IRE1α activation is regulated and fine-tuned by its regulatory partners both from the ER lumen and cytoplasmic side. In this section, considering IREα as the center molecule, we have discussed its partners and their role in different signaling events and how these can be mechanistically modified to orient cell towards death and survival. Apoptosis activation in response to ER stress may not be due to the preferential activation of a single UPR branch or by a switch from one branch to the other; rather, it could be due to the relative timing of IRE1, and PERK signaling determines the shift from cell survival to apoptosis [135].

## 8. IRE1α in Cellular Signaling: Calcium, ROS

The intracellular calcium ions regulate many cellular processes like exocytosis, transcription, cell proliferation, and apoptosis [165]. Usually, intracellular calcium levels are tightly regulated by multiple calcium channels, pumps, and binding proteins. Calcium released from intracellular stores of endoplasmic reticulum, mitochondria, lysosome, and nucleus eventually moves across the cell membrane to maintain the intracellular calcium concentration. Among these, ER is the most important. It can store calcium thousands of time higher the cytoplasmic calcium level [166].

Two calcium-release channels in the ER membrane are inositol 1,4,5-triphosphate receptors (IP3Rs) and ryanodine receptors (RyRs) [167,168], and the Ca^2+^ inlet channel consisting of sarco-endoplasmic reticulum Ca^2+^-ATPases (SERCAs) allows Ca^2+^ movements across the ER membrane [169]. In spite of tight regulation of Ca^2+^ release from the ER, several stress stimuli result in depletion of ER calcium and an overload of cytosolic calcium. The increased cytoplasmic calcium can trigger apoptosis through abnormal activation of calpain or phosphatase calcineurin in the cytoplasm [170,171], and activation of ER-resident caspases or mitochondrial dysfunction [172].

UPR sensor IRE1α has been shown to be involved in the regulation of calcium release through IP3R not by direct interaction, but with other adapter molecule apoptosis signal-regulating kinase 1 (ASK1). Usually, calcium and integrin binding protein 1 (CIB1) binds to IP3R and inhibits Ca^2^^+^ release from IP3R [173] and In addition, it is assumed that CIB1 calcium regulation is modulated by ASK1 interaction [174]. In SHSY5Y cells, knockdown of IRE1α results in more cytoplasmic calcium due to enhanced interaction of CIB1-ASK1 and free the IP3R from CIB1 inhibition. IRE1*α* regulates Ca^2^^+^ homeostasis of the ER by trapping ASK1 and reduces the binding of ASK1 and CIB1, and also reduces cell death due to the calcium-mediated ROS accumulation. IRE1α plays a role in ER calcium homeostasis in physiological and pathological conditions [175]. However, it is well known that the IRE1α-ASK1 pathway mediates cell death under pathological conditions [14]. Activation of IRE1α due to ER stress leads to dimer/oligomer, then depending on the stress level, IRE1α binds to TRAF2 and ASK1. In normal conditions, IRE1α mostly exists as a monomer, so interaction with TRAF2/ASK1 is questionable. Further studies are required to clarify how it will be different in normal/stress condition, whether it is in monomer/dimer state.

Furthermore, it is known that phosphorylation of Bcl-2 affects ER calcium homeostasis and also its antiapoptotic activity [176]. When Bcl-2 is phosphorylated, calcium discharge from the ER is increased with a secondary increase in mitochondrial calcium uptake. Low-level ER stress or preconditioning, surprisingly, increased the phosphorylation of Bcl-2 by IRE1α at Ser70, which exerts hepatoprotection through increased autophagy [148]. However, in another study, phosphorylated Bcl-2 showed decreased antiapoptotic activity due to decreased interaction with pro-apoptotic proteins [177]. In addition, the downstream target of IRE1α molecule JNK can phosphorylate Bcl-2 at Thr69, Ser70, and Ser87 within the unstructured loop [178,179]. Therefore, phosphorylation of Bcl-2 either directly by IRE1 or through JNK may have an impact on ER calcium homeostasis. These studies showed the significance of IRE1α in calcium homeostasis and cell survival during ER stress and revealed a previously unknown calcium-mediated cell death signal between the ER IRE1α-InsP3R pathway and the mitochondrial redox-dependent apoptotic pathway. In addition, the IRE1α/XBP1 pathway exhibits endoplasmic reticulum calcium store expansion and amplified calcium-mediated inflammation [180].

IRE1α is predominantly located in mitochondria-associated membranes (MAMs). The ER supplies calcium directly to mitochondria via IP3Rs at MAM [159]. Sig-1R interacting molecule with IRE1α translocates under chronic ER stress to MAM and influences IP3R [181], and stabilizes IRE1α to increase the prolonged activation of the IRE1α-XBP1 pathway, thus facilitating cell survival [182]. Therefore, the IRE1-Sig1R-IP3R complex may possibly have a role in the regulation of ER-mitochondrial interorganellar Ca^2+^ signaling and cell survival. The uptake of calcium in the mitochondrial matrix enhances oxidative phosphorylation as a cofactor of several TCA cycle metabolic enzymes [183]. A recent study shows that IRE1α’s contribution to preserving the structure and role of MAM in fine-tuning of mitochondrial respiration. The decrease in the rate of mitochondrial calcium uptake recorded here in IRE1α KO MEFs could translate into a drop in ATP levels, involving adaptive mechanisms to maintain cell survival, including the AMPK energy sensor, and catabolic processes such as autophagy induction [184]. Overall, this study indicates that, in the absence of ER stress, IRE1α has a household function in mediating ER-to-mitochondrion contact.

Reactive oxygen species (ROS) is the most prominent molecule involved in cell signaling. Imbalance in the ROS dynamics triggers cell death. This is produced usually through the electron transport chain and the oxidative protein folding in mitochondria and ER, respectively [185,186]. Additionally, ROS may also be generated as the primary function of NADPH oxidase (Nox) family enzymes [187]. It is well known that increased ROS in the cell results in the ER stress and UPR activation, but it is required to know that any downstream activities of the UPR signal transducers generate ROS. Here, we focused on activated IRE1α’s possible involvement in ROS generation. Increased cytosolic concentration of Ca^2+^induces mitochondrial ROS production [188]. IRE1α-deficient cells showed more ROS release from the mitochondria due to dysregulated calcium release from the ER, which results in increased calcium influx to mitochondria. IRE1α may be indirectly involved in the ROS generation through Ca^2+^- mediated signaling between the IRE1α-InsP3R pathway in the ER and the redox-dependent apoptotic pathway in the mitochondrion.

IRE1-dependent activation of CHOP through XBP1s and ASK1/p38 MAPK activation contributes to ROS generation [189,190]. Interconnected signals between ER and mitochondria are the main source of ROS. IRE1α triggered sustained activation of JNK, mediated the mitochondrial damage by binding to the outer mitochondrial membrane protein Sab (SH3 homology associated BTK binding protein) and subsequent inhibition of mitochondrial respiration [191], further leading to upstream activation of the mitogen-activated protein (MAP) kinase cascade and induce the cell death [192]. This could be very important in disease progression like in cardiovascular diseases like ischemia/reperfusion injury, neurodegenerative diseases, and inflammatory diseases.

IRE1-instigated ROS mediated by JNK may also influence the stem cell proliferation and also regulates intestinal stem cell (ISC) function and regenerative homeostasis in the intestinal epithelium [193]. IRE1α being a UPR molecule and able to interact with PDI, an oxidoreductase catalyzed disulfide bond formation and subsequent ROS [194]; thus, IRE1α and PDI interaction may have a role in ROS generation. RIDD activity of IRE1α generates ROS and oxidoreductase imbalance by increasing the thioredoxin interacting protein (TXNIP) through degrading TXNIP repressor microRNA miR-17, further inducing cell death [195]. ER stress is generated during a bacterial infection as a body defense mechanism. Though immune-secretory function is well established, the IRE1 pathway of ER stress can kill the bacterial pathogen by sustaining ROS generation through an NOX2-dependent manner [196].

ROS, such as hydroxyl radicals (OH), hydrogen peroxide (H2O2), and superoxide anion (O2-), are chemically reactive to various biological objectives [197]. Dynamic protein cysteine thiols oxidation by H2O2 leads to cysteine sulfenylation (SOH), sulfinylation (SO2H), and sulfonylation (SO3H). Among these, oxidation to SO3H is irreversible. S-sulfydration (also called persulfidation) can happen after responses between subsidiaries of hydrogen sulfide (H2S) and thiols [198]. Reactive nitrogen species (RNS) like nitric oxide (NO) react with some cysteines causing S-nitrosylation/nitrosation [199]. Developing evidence proposes that numerous proteins perhaps directed through cysteine adjustment. Previous observations appeared in *C. elegans*, and human cells that incorporated IRE1 have an unmistakable redox-regulated work in cytoplasmic homeostasis. ROS that are produced at the ER or by mitochondria sulfenylate, a cysteine inside the IRE1 kinase activation loop. This restrains the IRE1-mediated UPR ER and starts the p38/SKN-1(Nrf2) antioxidant reaction, thus expanding stress resistance and life expectancy [200]. In addition, in our in-vivo and in-vitro studies under chalcone (a natural anticancer agent) treated conditions, it was observed that ER-localized ROS sulfonate at a cysteine residue of IRE1α, by decreasing XBP1 splicing and increasing RIDD axis, thereby increasing cell death (unpublished).

## 9. Potential Role of IRE1α in Chronic Metabolic Diseases and Its Influence on Metaflammation

ER stress-mediated IRE1 signaling can generate a key inflammatory signaling pathway via JNK activation or other pathways, which can activate many inflammatory genes [14], which may lead to disrupting some metabolic function. Chronic low-grade metabolic inflammation or metaflammation [201] is a critical factor for type 2 diabetes and obesity-induced insulin resistance. Here, we describe about potential role of IRE1 in type 2 diabetes and obesity-induced insulin resistance influencing metaflammation.

### 9.1. Type 2 Diabetes

Diabetes is the major cause of morbidity and mortality in the modern era and has decreased both quality of life and life expectancy. Diabetes is a condition of abnormal blood glucose levels. Metabolic glucose uptake by the tissues is mainly dependent on the insulin and glucagon levels, which are majorly secreted from the pancreatic β-cells. Pancreatic beta cell’s endoplasmic reticulum has a huge task in terms of secretory protein folding in relation to the blood glucose level and plays a pivotal role in blood glucose homeostasis. Diabetes can be type 1 diabetes with an insufficient insulin level or it can be type 2 diabetes where tissues have insensitivity to insulin (insulin resistance). Type 1 diabetes is the result of loss of pancreatic beta cells due to the autoimmune destruction, and type 2 is defective in insulin-sensing cells as well as beta cell death. However, both conditions have been linked to the ER stress [202].

The onset of type 2 diabetes seems to be UPR activation. High blood glucose level induces beta cells to synthesize insulin. If persisting, this overwhelms the ER capacity and leads to the accumulation of misfolded protein. This disturbed ER environment induces beta cell impairment and consequently affects other cellular processes. In type 1 diabetes, the direct involvement of UPR is a little skeptical. However, recent studies reported the involvement of UPR in the destruction of beta cells.

IRE1 as a major UPR molecule plays a critical part in beta cell survival and function, and it has been involved in the homeostatic direction of pancreatic islet β-cells. Usually, pancreatic beta cells always experience ER stress to meet the insulin demand, but it will be physiological adaptive stress. However, in pathologic situations, ER stress exacerbates UPR sensor activation and then leads to abnormal cellular functions. The small variation between the physiological input of insulin translates into the ER and the folding capacity of the ER and disturbs the homeostasis of β cells, leading to ER stress [95]. Insulin biosynthesis is a key point in glucose metabolism. IRE1α plays a major role in insulin biosynthesis and in signaling through XBP1s and also maintains the oxidative balance in beta cells through RIDD activity [203]. IRE1α conditional knockout mice exhibited mild hypo-insulinemia, hyperglycemia, and a low-weight trend [93]. Furthermore, pancreatic-β-cell-specific IRE1α-conditional KO (cKO) mice and IRE1α-cKO insulinoma cell lines showed the requirement of IRE1α for the upregulation of insulin-folding enzymes to balance with insulin requirements [204].

Both transient and chronic high-glucose exposure of islets, INS1 cells, and mice activated the IRE1α. Glucose concentration normally fluctuated between 4 and 10 mM in the physiological state and treatment of islets with 5 and 10 mM glucose for 1 h increased IRE1α phosphorylation in a concentration dependent manner [95]. The high glucose-induced activation of IRE1 in an acute and chronic condition, showing a distinct downstream signaling mechanism of IRE1α. IRE1 does not have XBP1s, and BiP dissociation is phosphorylated in acute treatment in INS1 cells [95], but chronic hyperglycemia induces normal ER stress accompanied with XBP1s and BiP dissociation. However, some questions need to be cleared here, such as how IRE1 is phopshorylated without BiP dissociation, and if splicing of XBP1 does not occur, then it may be possible that IRE1α is activated, but it may be in dimer form since it was reported that dimer form induces RIDD rather than XBP1s. In addition, it may be possible to activate BiP-associated IRE1 under conditions of mild ER stress [38], a physiological regulatory mechanism by which the selective regulation of IRE1α kinase activity participates in a specific cellular function, which in this case is insulin biosynthesis. Additionally, severe high glucose stimulates interaction of receptor for activated C kinase 1 (RACK1) and protein phosphatase 2A (PP2A) to promote dephosphorylation of IRE1α, resulting in the attenuation of IRE1alpha activity and reduced insulin production [147]. In contrast, hyperactivated IRE1α degrades insulin mRNA and then suppresses insulin production [95]. Interestingly, IRE1α deletion in β cells increased the expression of inflammation and oxidative stress-related mRNA [203].

β-cell-specific XBP1 mutant mice caused hyperglycemia and glucose intolerance due to decreased insulin secretion from β-cells due to hyperactivated IRE1α which degraded a subset of mRNAs encoding proinsulin-processing enzymes and insulin mRNA through RIDD, contributing to the reduction of proinsulin biosynthesis and further β-cell death [205]. It suggests that IRE1α has dual and opposite roles in the function of β-cells and that a precisely controlled feedback circuit involving IRE1α and its product XBP1s is needed to achieve optimal insulin secretion and glucose regulation. IRE1/XBP1 contributes to adaptive response in beta cells that are exposed to high glucose conditions [206] and also promotes the compensatory proliferation of beta cells in the face of insulin resistance [111]. Furthermore, IRE1α facilitates diabetic wound healing by improving angiogenesis through degradation of angiogenic factors repressing miRNAs, miR-466, and miR-200 family members [207].

**Figure 1:**
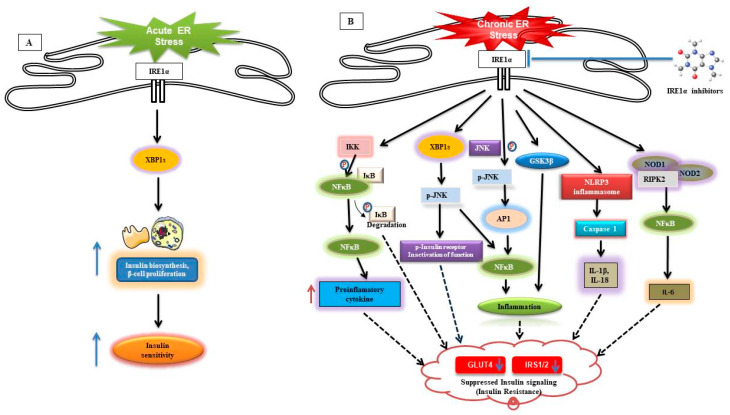
Possible mechanism of IRE1 in involvement of insulin signaling during acute and chronic Endoplasmic reticulum stress. (**A**) IRE1 α-XBP1s branch can generate cellular survival through increased insulin sensitivity during an acute or short-term ER stress condition. (**B**) However, over a long or chronic period of time, endoplasmic reticulum (ER) stress-, serine/threonine-kinase/endoribonuclease IRE1 α -binds to TNF receptor-factor 2 (TRAF2), apoptosis signaling kinase1 (ASK1), and receptor-serine/threonine protein kinase 1 (RIPK1), resulting in c-N-kinase phosphorylation this eventually triggers insulin receptor ablation and results in insulin resistance. C-Jun then interacts with c-Fos forms the active transcription factorAP-1, and increases IL-6 and TNFα production. In addition, the IRE1α/TRAF2/ASK1 complex activates the inhibitory kappa B kinase (IKK), which phosphorylates kappa B (IκB) inhibitor, leading to the release and translocation into the nucleus where cytokine expression is induced. Proteasomes then degrade the dissociated IκB. The IRE1α–TRAF2 complex increases IL-6 production through the combination of the nucleotide-oligomerization domain (NOD)-containing proteins 1 and 2 (NOD1 and NOD2) and serine/threonine-kinase 2 (RIPK2) receptor-complex. IRE1α produces splices via its RNase function—X-box-binding protein 1 (XBP1s) transcription factor induces several pro-inflammatory cytokine expression. However, XBP1s improves nuclear translocation by mediating the degradation of FoxO1, an NFκB inhibitor. In addition, the activation of IRE1α differentially controls the expression of the pro-inflammatory cytokine IL-1β gene by glycogen synthase kinase-3β activation. The controlled IRE1α-dependent decay (RIDD) degrades miR-17, resulting in increased expression of the protein that interacts with thioredoxin. This triggers the nucleotide-binding domain, leucine-rich-containing family, pyrin domain-containing-3 inflammasome activity, leading to procaspase-1 cleavage, which subsequently activates IL-1β and IL-18. Production of this all pro-inflammatory cytokines and inflammatory response through IRE1 either directly or indirectly leads to insulin resistance by the inhibition of insulin signaling and the activation of gluconeogenic enzymes. In addition, it may be possible to reduce the development of insulin resistance by inhibiting either small chemical molecules such as KIRA6/KIRA8, STF-083010, MKC3946, MKC8866, MKC9989, B-I09, A-I06, 4μ8C Sunitinib, Imatinib, Fortilin.

IRE1α looks essential for insulin biosynthesis after glucose stimulation in pancreatic beta cells in both XBP1-dependent and -independent manner (Figure 1). However, under chronic metabolic stress, IRE1α is implicated in the progression of diabetes and its related complications like cardiomyopathy, retinopathy, nephropathy, and neuropathy. It is interesting to know whether IRE1α activation results in diabetes or diabetic condition activates IRE1α. The precise role of IRE1α in integrating metabolic ER stress signals to regulate β-cell functions still needs to be investigated. Mice fed with a high-fructose diet developed hepatic insulin resistance due to inhibition of insulin-mediated Akt phosphorylation by IRE1-JNK pathway and diet-impaired hepatic insulin signaling (Figure 1B) [208].

Recent evidence indicates that both saturated fats and inflammatory mediators such as cytokines trigger UPR in pancreatic beta cells. IRE1/XBP1 pathway potentiates the activation of nuclear factor κB, a key regulator of inflammation, exposes the beta cells to the proinflammatory effects of cytokines. This can contribute to the upregulation of local inflammatory mechanisms and aggravation of insulitis. The dialogue between the UPR and inflammation may provide an explanation for the parallel increase in the prevalence of childhood obesity and type 1 diabetes.

Especially in obesity-induced high blood glucose due to insulin resistance, XBP1s upregulate the cyclin D1, which is required to drive cells from G1 into the S-phase of the cell cycle [112] and to promote the compensatory proliferation of β-cells (Figure 1A). Furthermore, persistence excessive ER stress disrupts the IRE1α-XBP1s-cyclin D1 pathway, which results in beta cell death [111]. IRE1 activity should be optimally regulated in situations of metabolic stress due to the overproduction of XBP1s that is deleterious to β-cell functions through inhibition of insulin, Pdx1, and Mafa expression, eventually leading to beta-cell apoptosis [209]. A recent study showed that IRE1 reduces glucose metabolism as part of an adaptive response [210].

IRE1α may exacerbate diabetic retinopathy because it is known to get hyperactivated during the hyperglycemic condition and may degrade the miRNAs and increase the stability of a pro-oxidant and pro-apoptotic TXNIP [195]. TXNIP has been associated with ROS/RNS stress, mitochondrial dysfunction, inflammation, and premature cell death in diabetic retinopathy (Figure 1B) [211]. In a high-glucose state, the expression of miR-17 is triggered and suppressed by IRE1, which leads to an increase in its target gene TXNIP (thioredoxin-interacting protein). High glucose-TXNIP increased its binding to the inhibitor ASK1, thioredoxin (Trx), and thus sequestered Trx from the Trx-complex. Glucose caused high activation of ASK1 and consequent apoptosis [212].

IRE1α may also contribute to maternal diabetes-induced ER stress in the developing embryo and cause embryopathy through ASK1-mediated JNK activation [213]. Downregulation of XBP1s and phosphorylation of IRE1α by Moutan Cortex reduce diabetic nephropathy and also showed decreased inflammatory molecules IL-6, MCP-1, and ICAM-1 expressions [214]. Expression of spliced XBP-1 varied in different experiment conditions [215,216]. However, sXBP-1 promotes cell survival, but prolonged stress attenuates the IRE1α/XBP-1 arm of the UPR, sensitizing cells to apoptosis [42]. Thus, regulation of IRE1α/XBP-1 pathway may slow or prevent the progression of diabetic complications. IRE1α-mediated CHOP and JNK activation induce apoptosis of beta cells in type 1 and type 2 diabetes [206]. Diabetic cardiomyopathy: IRE1α triggered JNK is also involved in the progression of cardiovascular diseases associated with obesity and diabetes [217].

### 9.2. IRE1α Contribution in Obesity-Induced Insulin Resistance and Metaflammation

Obesity is a major complication in the modern world. Excess accumulation of fat in different tissues integrates the metabolism and inflammation, causes chronic low-grade inflammation or metaflammation majorly in metabolic tissue, and then causes problems in multiple sites [218]. Generally, this interaction tries to bring metabolic homeostasis, but the disturbance in this association due to mediators produced from the interface leads to a progression of immunometabolic disease and premature cell death. Obesity is usually characterized by pro-inflammatory cytokines, free fatty acids, and high blood glucose [219]. It has been linked to many disorders including cardiovascular diseases, insulin resistance, type 2 diabetes, inflammatory disease, and many more. An important primer for metaflammation is chronic overloading of the endoplasmic reticulum (ER) and consequent stress. Obesity induces the ER stress in adipocytes, hepatocytes, macrophages, pancreatic beta cells, and neurons. However, ER stress-mediated obesity-related complications may vary depending on the tissue environment.

One of the mechanisms which related to different complications is ER stress-mediated UPR activation. Among the UPR molecules, IRE1α contributes considerably to the progression of these diseases. In both genetic and diet-induced models of obesity, IRE1α is prominently activated [220]. The IRE1α/XBP1 pathway contributes significantly in lipogenesis through the transcriptional induction of lysogenic genes. Xbp1+/− mice exhibit increased ER stress coupled with impaired glucose and insulin tolerance in the high fat diet (HFD)-induced obesity [221], but in pathological manifestations, activated IRE1α modulates many downstream molecules which consequently result in disease progression. IRE1α in chronic stress phosphorylates JNK, and the phosphorylated JNK affects the glucose uptake in the cells through phosphorylating insulin receptor “known to inactivate the function” (Figure 1B). The absence of JNK reduced adiposity, substantially enhanced insulin sensitivity, and increased signaling ability of insulin receptors in mice [222,223]. This insulin resistance results in a hyperglycemic condition, which increases the burden on pancreatic beta cells to produce more insulin and consequently, ER stress develops, leading to the development of type 2 diabetes due to beta cell loss [186]. Furthermore, obesity induces chronic low-grade inflammation, which also negatively impacts insulin sensitivity [224,225].

IRE1α is one of the key UPR transducers in the pathogenesis of obesity-related inflammation by activating cytokine transcription factor AP-1 through JNK and by increasing the NF-κB nuclear translocation through promoting degradation of IκB by IKK-mediated phosphorylation [226,227]. Additionally, phosphorylation of JNK and IκK is known to impair insulin action and glucose homeostasis [228,229]. Insulin resistance also reduces XBPs nuclear translocation by interfering with PI3K dimer disruption, then worsens the ER stress [230], but, contrastingly, disrupted PI3K can also potentiate the JNK-mediated insulin resistance (Figure 1B) [231]. Increased JNK and NF-κB signaling influences pro-inflammatory cytokine synthesis and In addition, NF-κB activation itself activates ER stress by a feed-forward loop, thereby maintaining an inflammatory state [232]. Furthermore, NF-κB activation produces inflammatory cytokine TNF-α, which impairs IRE1α-deficient mouse embryonic fibroblasts (MEFs) [227], but overexpression of XBP1 subsequently blocks the IRE1α/IKK/NF-κB pathway [233].

Generally, XBP1s are important for metabolic homeostasis, and the liver of ob/ob mice showed increased nuclear XBP1s protein levels [234]. However, interestingly, XBP1 absence in the liver protected against insulin resistance [235]; in contrast, another study documented that XBP1s functions as an anti-lipogenic factor through suppression of genes involved in the synthesis of hepatic triglyceride and diacylglycerol in livers of diet-induced obese and insulin-resistant ob/ob mice and also by enhancing lipolysis [236]. Additionally, in metabolic disorders, IRE1α also activates the GSK-3β, a major regulator of peripheral inflammatory responses, mediates the pro-inflammatory cytokines IL-1β and TNF-α through downstream molecules and XBP. In contrast, the activation of GSK-3b inhibited the splicing of XBP-1, resulting in the downregulation of TNF-α production (Figure 1B) [237]. Furthermore, obesity-mediated iNOS and nitric oxide cause insulin resistance by s-nitrosylating the IRE1α, which affects the ER homeostasis role by inhibiting the XBP1 splicing, but maintaining the IRE1α phosphorylation and c-Jun N-terminal kinase (JNK) activation and its mediated inflammation [238].

IRE1α in adipose tissue-recruited macrophages (ATMs) distinctly contributed to the obesity-associated inflammation. M1 macrophages are hallmarks of obesity-associated inflammation within white fat. Macrophage-specific deletion of IRE1α reduced the high-fat diet-induced hepatic steatosis, insulin resistance, and also pro-inflammatory cytokines IL-1β or TNF [239]. It is also possible that excess fatty acid-activated Toll-like receptors (TLR) can induce the IRE1α/XBP1 inflammatory cytokine production in macrophages [240]. For example, in pseudomonas bacterial infection, the TLR-induced IRE1α–XBP1 cascade mediated by ROS produced the pro-inflammatory cytokines IL-6 and TNFα required for host defense [241].

High-fat-diet/obesity-mediated ER stress triggers the pattern recognition receptors NOD1/2 mediated inflammation, which contributes to the development of type 2 diabetes [242,243]. A recent study reported that thapsigargin and dithiothreitol-induced ER stress trigger the production of the pro-inflammatory cytokine IL-6 in an IRE1α/RIP2/NOD1/2-dependent fashion. IRE1α kinase inhibitor application attenuated the NOD1 and NOD2 mediated pro-inflammatory responses [244]. Two small inhibitor molecules, STF-083010 and 4µ8C, which selectively inhibit the RNase function of IRE1α, in an application study in atherosclerosis, which is the best example of metaflammation disorder. These IRE1α inhibitors decreased hyperlipidemia-induced IL-1β and IL-18 production, lowered T-helper type-1 immune responses, and reduced atherosclerotic plaque size [130], although the above evidence showed a great deal of variation on the experimental system used. Obesity-mediated IRE1α contributes in the low-grade inflammation, metaflammation, in metabolically critical organs and leads to insulin resistance and subsequent type 2 diabetes. Optimized targeting like neither constitutive activation nor complete inhibition of RNase/kinase activity of IRE1α itself or disruption of its downstream molecule interaction will be a possible therapeutic option in controlling chronic disease. IL-1β is a significant contributor to the inflammation, insulin resistance caused by obesity, pancreatic β-cell dysfunction, and type 2 diabetes. IRE1 also contributes to the lipid-induced activation of NLR family pyrin domain containing 3 (NLRP3) inflammasome, a multicomponent complex that contains caspase-1 and induces the caspase-1–dependent secretion of the pro-inflammatory cytokines IL-1β and IL-18 [245,246]. Furthermore, inhibition of NLRP3 inflammasome protected the pancreatic β-cells from cell death during obesity and progression of type 2 diabetes [247]. IRE1α/XBP1 activation can also inhibit the IRS1/2 signaling through inducing P300 acetyltransferase involved in glucose production, then promoting the insulin resistance in obese mice [248,249].

## 10. Conclusions

Collectively, available information through recent investigations suggested that the IRE1 plays a significant role in cellular fate in various physiological and pathological conditions. During physiological processes such as divergent cell fate and metabolism, understanding the structure and its mode of activation enables us to describe its potential influence on the homeostatic balance/maintenance, a core of physiologic process. Under the pathological conditions such as nutrient dysmetabolism and disease-designated diabetes, the modulation of IRE1α activity is suggested to be a therapeutic strategy to control the pathologic state. Therefore, its applicability needs to be widened for therapeutic benefits. Being an ER stress sensor, IRE1 needs to be understood from a wider perspective, not restricting to structure or mode of action. Thus, it is necessary to apply the understanding of IRE1 to elucidate its biological meaning and assemble the future needs with regard to pathological conditions arising from UPR activation and ER stress.

## Figures and Tables

**Table 2:** Partners in regulating IREα endoribonuclease activity.

IRE1α Binding Partner	Function of IRE1α Binding Partner	Functional Implication	References
NMIIB (Non muscle myosin IIB)	A Cytoskeleton myosin protein	Interacts with IRE1α and regulates its oligomerization and activation. In addition, recruits other regulatory molecules to oligomerized foci.	[136]
AIP1	Apoptotic signaling transducer	AIP1-IRE1α association enhances IRE1 dimerization and its downstream JNK/XBP1 activation.	[137]
PDIA6	Chaperonic protein of ER that inhibits aggregation of misfolded proteins	PDIA6 attenuates the activity of IRE1α. PDIA6, an ER resident protein disulfide isomerase. Negatively regulates IRE1α by binding to its luminal domain at cysteine 148, if it is oxidized, IRE1α will be activated. PDIA6-deficient cells hyperrespond to ER stress with sustained autophosphorylation of IRE1α and increased XBP1s, pJNK.	[138]
PTP-1B	Protein-tyrosine phosphatase 1B	In the absence of PTP-1B, ER stress-induced IRE1α downstream activities were impaired, especially XBP1 splicing and JNK activation.	[139]
UbD	Ubiquitin-like modifier family member	UbD regulates IRE1α/c-Jun N-terminal kinase signaling pathway. It provides a negative feedback on cytokine-induced activation of the IRE1α/JNK pro-apoptotic pathway in cytokine-exposed beta cells, but did not change cytokine-induced XBP1 splicing.	[140]
TMBIM6	ER localized antiapoptotic protein, also known as Bax inhibitor-1 (BI-1)	This has been implicated in the negative modulation of XBP1 splicing activity through interacting with a cytosolic region of IRE1α.	[141]
Hsp47	Heat shock protein	Hsp47 binds directly to the IRE1 ER luminal domain with high affinity, eliminating BiP from the complex to allow IRE1α oligomerization for optimal signaling.	[142]
HSP72	Heat shock protein	Overexpression of HSP72, survival effect of HSP72 under ER stress is mediated by enhanced XBP1splicing and its target genes. Regulation of UPR by HSP72 is by formation of stable protein complex with IRE1α.	[143]
HSP90	Heat shock protein	HSP90 stabilizes IRE1α by preventing the proteasomal degradation, and treatment of HSP90 inhibitor decreases IRE1α protein stability.	[144]
JIK	c-Jun N-terminal inhibitory kinase	IRE1α and TRAF2 complex induce apoptotic signal through c-Jun N-terminal kinase pathway and activation of caspase-12.	[145]
JAB1	Jun activation domain-binding protein-1	Mutant JAB1 down-regulates the UPR signaling pathway through tight binding with IRE1alpha.	[146]
RACK1	Receptor for activated C-kinase 1	Interacts with IRE1α and plays a role in dephosphorylation of IRE1α by protein phosphatase (PP2A). Furthermore, IRE1α and RACK1 association may contribute in this process of antiapoptosis by phosphorylating AMPK and Bcl-2 through enhancing autophagy.	[147,148]
Nck	SH2/SH3 adaptor protein	Nck and IRE1α association in immune T cells have a critical role in ER-stressed activation of MAPK pathway and cell survival.	[149]
RNF13	RING finger protein	RNF13 knockdown cells showed resistance to apoptosis and JNK activation triggered by ER stress. Conversely, overexpression of RNF13 induces JNK activation and caspase-dependent apoptosis.	[150]
PARP16/ARTD15	Poly ADP-ribose polymerases/ADP-ribosyl transferase D proteins	PARP16 is an upstream regulator, and modification increases its kinase and the endonuclease activity of IRE1α.	[151]
BAX/BAK	Pro-apoptotic protein	BAX and BAK directly interact at cytosolic domain of IRE1α during stress condition and promote the stabilized IRE1α activity.	[152]
BIM/PUMA	Pro-apoptotic protein	BIM and PUMA have also been linked to IRE1α regulation by direct binding with IRE1α via their BH3 domain in stress-dependent manner. Cells deficient in both BIM and PUMA exhibited reduced splicing of XBP-1 and RIDD.	[153]
NMI	N-Myc interactor	Interacts and modulates IRE1α especially in pancreatic beta cell. It negatively regulates the IRE1α-mediated JNK activation and further the cell death.	[154]
DCR2	Dose-dependent cell-cycle regulator 2	Physically interacts with phosphorylated IRE1α and causes dephosphorylation and IRE1 deactivation.	[155]
Cab45S	A member of the CREC family	Negatively regulates RNAse activity of IRE1α and prevents more spliced forms of X-box-binding protein 1 mRNA at the early stage of stress and further phosphorylation of c-Jun N-terminal kinase induced apoptosis.	[156]
SYVN1	Functions in ER-associated degradation process	Coexpression of IRE1 and SYVN1 increased IRE1 degradation and ubiquitination.	[157]
DDRGK1	DDRGK domain-containing protein 1	Interaction of DDRGK1 with IRE1α counteracts ubiquitination and subsequently inhibits the ERAD-mediated degradation of IRE1α.	[55]
PRKCSH	Protein kinase C substrate 80K-H	In ER stress condition, PRKCH steps up ER stress-mediated autophosphorylation and oligomerization of IRE1 through mutual interaction followed by XBP1 splicing and MAPK activation which contribute to tumorigenesis.	[158]
Sigma-1 receptor	Unique ligand-regulated molecular chaperone in the ER.	Under ER stress conditions, interacts with and stabilizes IRE1α and enhances cell survival through prolonged activation of the IRE1α-XBP1 pathway, especially in cancer cell survival.	[159]
Sec61	Channel-forming translocon complex	Forms a hetero-oligomeric complex with IRE1α upon ER stress. It recruits XBP1u and aids in splicing. The Sec61-IRE1α complex defines the extent of IRE1α activity and may determine cell fate decisions during ER stress conditions.	[160,161]
Fortilin	Pro-survival molecule	Interacts with the cytoplasmic domain of IRE1α, inhibits both kinase and RNase activities, and protects cells from apoptotic cell death.	[162]
Filamin A	Actin crosslinking factor involved in the remodeling of cytoskeletons	Through a novel domain located at the distal C-terminal region, monomeric IRE1α interacts physically with Filamine A. A pro-migratory stimulus causes dimerization of IRE1α, increasing Filamin A binding and PKCα recruitment. Phosphorylation of Filamine A by PKCα at S2152 improves actin cytoskeleton remodeling and cell migration in different animal species	[163]
ABL kinase	Tyrosine-protein kinase	ABL kinase interaction enhances IRE1α RNase activity and potentiates its apoptosis signaling pathway.	[164]

## Abbreviations

ATP	Adenosine triphosphate
ASK1	Apoptosis signaling kinase 1
ATF6	Activating transcription factor 6
ATF4	Activated transcription factor 4
ATMs	Adipose tissue-recruited macrophages
BiP	Binding immunoglobulinprotein
CSSR	Core stress-sensing region
CHOP	C/EBP homologous protein
CIB1	Calcium and integrin binding protein 1
DTT	Dithiothreitol
DR5	Death receptor 5
ER	Endoplasmic reticulum
eif2α	Eukaryotic translation initiation factor 2 alpha
ERAD	Endoplasmic-reticulum-associated protein degradation
GRP78	Glucose-regulated protein 78
IRE1α	Inositol-requiring transmembrane kinase endoribonuclease-1α
IP3Rs	Inositol 1,4,5-triphosphate receptors
ISC	Intestinal stem cell
JNK	c-Jun N-terminal kinase
KIRAs	Kinase-Inhibiting RNase Attenuators
MHCs	Major histocompatibility complexes
MAMs	Mitochondria-associated membranes
MTP	Microsomal triglyceride transfer protein
MTORC1	Mammalian target of rapamycin complex 1
NLRP3	NLR family pyrin domain containing 3
MEFs	Mouse embryonic fibroblasts
ORF	Open reading frame
PP2A	Protein phosphatase 2A
PP1C	Protein phosphatase 1C
PERK	PRKR-like endoplasmic reticulum kinase
PHLDA3	Pleckstrin homology-like domain family A, member 3
ROS	Reactive oxygen species
RyRs	Ryanodine receptors
RACK1	Receptor for activated C kinase 1
RIDD	Regulated IRE1-dependent decay
SERCAs	Sarco-endoplasmic reticulum Ca^2+^-ATPases
TXNIP	Thioredoxin interacting protein
TLR	Toll-like receptors
TRAF2	TNF receptor-associated factor 2
UPR	Unfolded protein response
XBP1	X-box binding protein 1
